# Being an Only or Last-Born Child Increases Later Risk of Obesity

**DOI:** 10.1371/journal.pone.0056357

**Published:** 2013-02-20

**Authors:** Line K. Haugaard, Teresa A. Ajslev, Esther Zimmermann, Lars Ängquist, Thorkild I. A. Sørensen

**Affiliations:** 1 Institute of Preventive Medicine, Frederiksberg and Bispebjerg University Hospitals, Frederiksberg, Denmark; 2 Novo Nordisk Foundation Center for Basic Metabolic Research, Faculty of Health and Medical Sciences, University of Copenhagen, Copenhagen, Denmark; University of Cincinnati, United States of America

## Abstract

**Background:**

Studies have suggested that number of siblings and birth order is associated with obesity. However, studies combining these exposures are needed. This study aimed at investigating obesity in children and young adults in regard to different combinations of family size and birth order.

**Methods:**

Two cohorts selected from the general population were investigated: The Copenhagen School Health Records Register (CSHRR) and a Draft Board (DB) sample with measured heights and weights in childhood (age 13 years) and young adulthood (age 19 years), respectively. Information on birth order, number of siblings, and relevant covariates were available on 29 327 children, as well as on 323 obese young men and 575 randomly selected controls of young men representing approximately 58 000. The relation between number of siblings and birth order, respectively, and having a Body Mass Index (BMI) z-score above or equal to the 95^th^ percentile in childhood or having a BMI of at least 31.00 kg/m^2^ in young adulthood was analysed using logistic regression analyses adjusted for relevant confounders.

**Results:**

Only children had significantly higher odds of obesity both in childhood and in young adulthood compared with children with siblings, odds ratio (OR) = 1.44 (95% Confidence Interval (CI): 1.26–1.66) and OR = 1.76 (95% CI: 1.18–2.61), respectively. No association between first-born status and obesity was found. The OR of last-born children being obese was also significantly increased in childhood, e.g. OR = 1.93 (95% CI: 1.09−3.43) of obesity if last-born in a family of four children. This was not found in young adulthood. Additionally, higher spacing to previous sibling (average 1872 vs. 1303 days; p = 0.026 in four children families) was observed in obese last-born compared to non-obese last-born children.

**Conclusion:**

Being an only or last-born child is associated with obesity. These associations may provide leads to targeted prevention of obesity in children.

## Introduction

Obesity in child- and adulthood has been recognized as a major public health problem worldwide [1]. Obese children often become obese adults; thus prevention of obesity from early ages is important [1]. The origin of obesity in children and young adults is complex and multiple factors including genetic, social and behavioural all seems to interact [1]. The family environment is a factor which may influence obesity risk, but which may also differ by family size and birth order.

Some of the earliest studies of family structures and obesity were conducted by the psychiatrist Hilde Bruch in the 1940s. Through direct observations on parents from child guidance clinics, certain features of maternal behaviour were considered the causal explanation of the child’s obesity. For both only and last-born children it was found that maternal ambivalence was associated with overfeeding and obesity [2]. A recently published study by Hunsberger *et al.* [3] also found that only children were uniquely at risk for obesity. However, the study did not distinguish between only and first-born children, and investigations of differences along birth order status and family size were not performed. Other studies have suggested that having siblings may be protective against development of overweight and obesity [4], [5], [6] but the evidence is still sparse. Furthermore, it is unknown whether the spacing between births has an influence on this. A few studies have examined the effect of birth order on overweight and obesity, but with inconsistent results [4], [5], [7]. It has been observed that first-born children carry a higher risk of metabolic disease such as hypertension [8], but without considering possible differences between first-borns who stay an only child and those who have siblings.

Since societies and family structures are changing world-wide [9] and subfecundity is increasing [10], a growing proportion of one-child families will most likely be seen in the future. If only children are at increased risk of obesity, and if not due to their first-born status, they may be an important at-risk group for targeted prevention. Furthermore, obesity risk may also differ along other groups of birth order and family size such as in last-born children as observed by Hilde Bruch [2] and in a recently published Japanese study [11]. The aims of this study were to examine the combined exposures of family size and birth order and obesity status in childhood as well as in young adulthood.

## Materials and Methods

### Population

Data originated from two large cohorts selected from the general population: The Copenhagen School Health Records Register (CSHRR) and The Draft Board (DB) examinations of young men. Obesity in childhood (13 years) was assessed through the CSHRR. The original school health examinations were conducted in all schools, whether public, private or specialized, in the Copenhagen municipality since 1936 and hold childhood BMI measures as well as other information subtracted into the case-cohort sample of young adult men [12], [13], [14]. However, obesity in these young adult men (median age 19 years) was investigated using BMI measures from the DB examinations.

**Figure 1:**
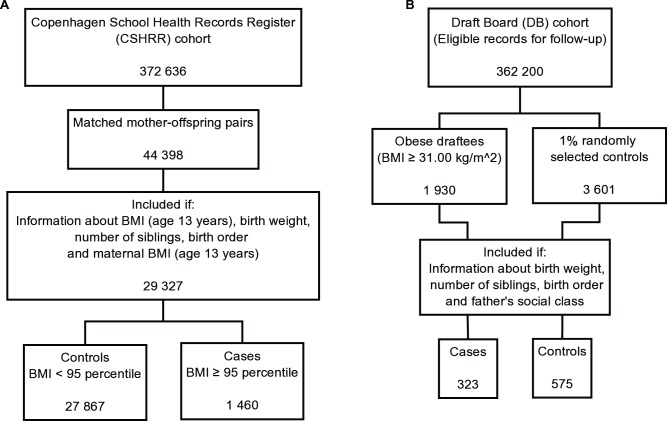
Flow-charts of the two sample selections based on eligibility and exclusion criteria.

By using the Central Personal Registration (CPR) numbers, which are assigned to all Danish citizens born after, or being alive at, the beginning of year 1968 and onwards, it was possible to link 329 632 of the 372 636 children in the CSHRR to the Fertility Database [15] through Statistics Denmark, which includes information from the birth registry as well as demographic registers. Thereby, information on family size and birth order within the CSHRR could be obtained. A sample of 28 709 mothers and 44 398 biologically related children, born between 1952 and 1989, was identified, and measured heights and weights at age 12–14 years as well as information on other covariates were available on 29 327 children (Figure 1a). Sex- and age specific BMI z-scores were generated using an internal reference population of children from years 1955–1960 [15]. Data from these years were used because the prevalence of overweight was low and stable during this period. BMI z-scores were calculated by subtracting BMI for each child from mean BMI in this fixed reference population and dividing the result with the standard deviation in the reference population. The z-scores were interpolated, assuming linear growth to age 13 years if two measurements between the ages 12–14 years were available or carried forward/backward if only one measurement was available within this interval [16]. Cases were identified as having a sex specific BMI z-score above or equal to the 95^th^ percentile at age 13 years. Complete information on maternal BMI from age 13 years, birth order, family size, birth weight and maternal age at child birth, was found on 1 460 obese and 27 867 non-obese children.

The second study population is a case-cohort sample (Figure 1b) drawn from an obesity study of 362 200 Danish young men undergoing DB examinations in the greater Copenhagen area and in an adjacent provincial area during the periods 1943–1977 and 1964–1977, respectively [13], [14], [17]. The DB examination was mandatory during the study period and was usually carried out between the ages of 18 and 26 years (median age 19 years). Height and weight (without shoes and in underwear only) were measured at examination time [13]. The DB cohort was established in the 1970’s. All 362 200 DB examination cards were screened manually to identify cases. The case-criterion threshold of a 35% excess weight with respect to the used Scandinavian standards was chosen [12], [17]. Initially, potential cases were identified if their weight in kilos exceeded or equalled their height in centimetres minus 80, which could be done through an easy head calculation. Afterwards when calculating the body mass index and computerizing data, it turned out that all members of the collected case series of obese men all had a BMI ≥31.00 kg/m^2^ (n = 1 930) and this threshold was therefore chosen to be the case-definition (the sampling criterion did not allow for defining the case samples by BMI ≥30 kg/m^2^). A 1% control sample (n = 3 601) was extracted [18]. School health records from the central Copenhagen municipality, which only constitutes a small part of the draft board region, were manually retrieved from the archives and information on birth order, number of siblings and paternal occupation, reported at school entry, was noted. Complete information from school health records was available on 323 of the obese and 575 of the randomly selected men. The sampling design implies that these 575 controls represent approximately 58 000 draftees, among whom the 323 cases were the most obese.

### Covariates

The following covariates were identified *a priori*: Year of birth (linear) and birth weight (linear) were included in both cohorts [19], [20]. Maternal age, maternal pre-pregnancy BMI [1] (using maternal BMI at age 13 years as a proxy) and birth spacing between siblings were only available in the analyses of childhood obesity. Social class was only available in the analyses of obesity in young men; it was defined by the fathers’ work position and categorized into three groups (1- unskilled & semi-skilled worker; 2- skilled worker, subordinate clerk and skilled worker with own business; 3- sub-academic, academic and advanced academic profession).

### Statistical Analyses

**Figure 2:**
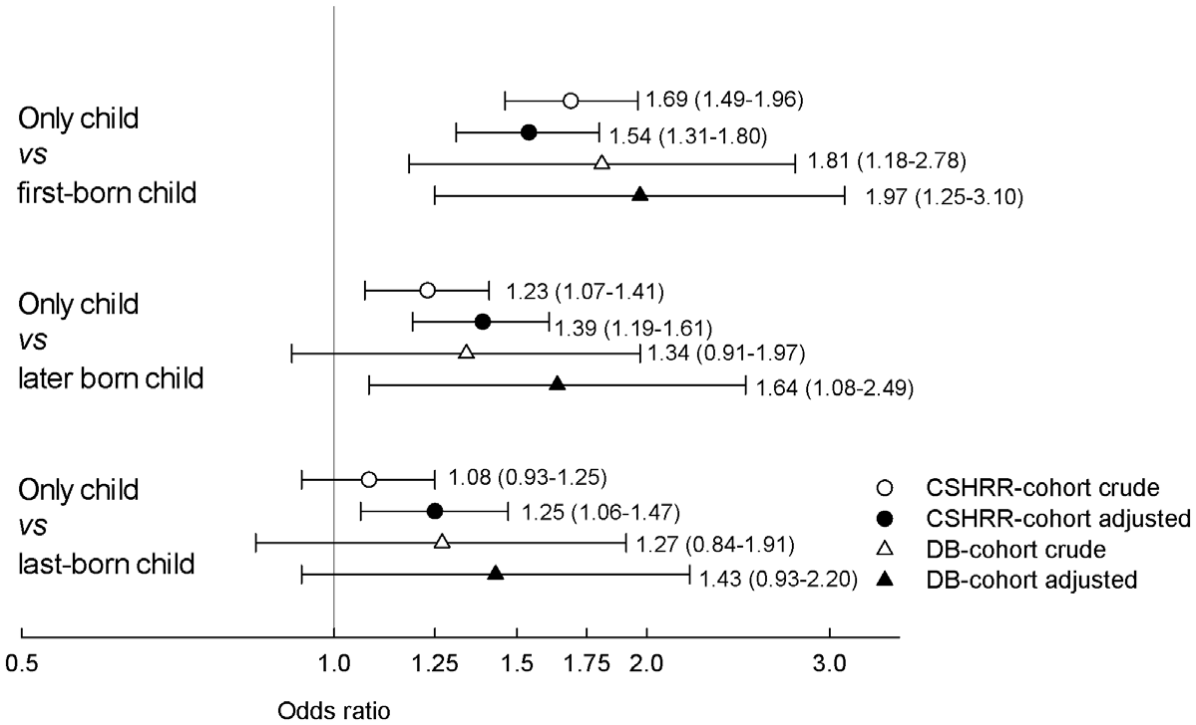
Odds of obesity by only child status vs. first-, later and last-born child, respectively. Crude and adjusted results (Odds ratio (95% CI)) for the Copenhagen School Health Records Register (CSHRR) cohort*^1^* as well as the Draft Board (DB) cohort*^2^* are shown, respectively. The adjusted results are adjusted for birth weight, birth year, *^1^*sex, *^1^*maternal age, *^1^*maternal BMI (age 13 years) and *^2^*father’s social class.

BMI distributions and covariates were summarized for the obese and the non-obese individuals, respectively by birth order (first, second, third, fourth, or fifth or more) and family size (one, two, three, four, or five or more children) (Table 1). Changes in the distributions of family size were investigated in childhood by two periods of birth year (1952–70 and 1971–89). With logistic regression analyses, we modeled the odds of being obese defined as having a BMI z-score above or equal to the 95^th^ percentile in childhood or having a BMI of at least 31.00 kg/m^2^ in young adulthood, in only, first-, and last-born children (as the exposed). Only children were excluded from the analyses of first- and last-born children. The odds of obesity in only children was compared with, respectively, children with any siblings (including families >4 children), and children having, respectively, one, two and three siblings (reference groups), as shown in Table 2. The odds of obesity in only children were also compared with the odds in first-, later and last-born children, respectively (reference groups), as shown in Figure 2. The odds of obesity in first-born children were compared with the odds in any later born children (families >4 children also included) as well as separately in families with, respectively, two, three or four children (reference groups), as shown in Table 3. Finally, we investigated the odds of being obese in last-born children compared with prior born children (excluding only children) as well as with prior born children in families with two, three or four children (reference groups), respectively, as shown in Table 4.

**Table 1:** Characteristics of the two study populations The Copenhagen School Health Records Register (CSHRR) and The Draft Board (DB) given as median (5–95% interval) and in percentages.

	CSHRR		DB	
	Cases (BMI ≥95^th^ percentile n = 1 460)	Controls (BMI <95^th^ percentile n = 27 867)	Cases (BMI ≥31kg/m^2^ n = 323)	Controls (random sample n = 575)
**BMI (kg/m** 2 **)**	25.3 (23.4–30.6)^1^	18.0 (15.2–22.1)^1^	32.7 (31.1–40.4)^2^	21.3 (18.3–25.9)^2^
**Family size**	**%**	**%**	**%**	**%**
1 (only child)	21.5	16.2	18.3	13.0
2	47.1	48.3	40.6	45.4
3	21.4	25.4	20.7	26.6
4	7.9	7.5	11.8	7.5
5+	2.1	2.7	8.7	7.5
**Birth order**	**%**	**%**	**%**	**%**
1	54.5	58.1	43.3	45.7
2	33.3	32.3	31.0	33.2
3	9.6	8.4	14.6	12.2
4	2.6	1.2	7.1	4.9
5+	0.41	0.35	4.0	4.0
**Birth weight (g)**	3500 (2500–4350)	3300 (2350–4200)	3500 (2500–4750)	3500 (2500–4500)
**Year of birth**	1974 (1958–1988)	1966 (1956–1986)	1948 (1939–1956)	1946 (1937–1955)
**Birth spacing (days)** 3 **Mother’s BMI (kg/m** 2 **)**	1624 (1033) 20.6 (2.9)	1299 (868) 18.6 (2.3)	–	–
**Mother’s age (years)**	25.4 (4.8)	24.2 (4.4)	–	–
**Father’s social class**	**%**	**%**	**%**	**%**
Unskilled workers	–	–	46.8	32.2
Skilled	–	–	49.9	61.4
Academic	–	–	3.4	6.4

1 at age 13 years,

2 at draft board examination (∼19 years),

3 between (consecutively born) sibling.

**Table 2:** Odds ratios (OR) and 95% confidence intervals (CI) for odds of obesity in only children by family size**.**

	Copenhagen School Health Records Register (Obese: ≥95^th^ percentile)						Draft Board (Obese: BMI ≥31.00 kg/m^2^)					
	Crude			Adjusted^1,2^			Crude			Adjusted^1,3^		
Only children vs.	OR	95% CI	p-value	OR	95% CI	p-value	OR	95% CI	p-value	OR	95% CI	p-value
Children with any siblings*(n_1_ = 4 823+24 504, n_2_ = 134+764)	1.42	[1.25–1.62]	<0.001	1.44	[1.26–1.66]	<0.001	1.49	[1.03–2.16]	0.036	1.76	[1.18–2.61]	0.005
Children in families with 2 children (n_1_ = 4 823+14 142, n_2_ = 134+392)	1.36	[1.18–1.57]	<0.001	1.38	[1.19–1.60]	<0.001	1.57	[1.05–2.34]	0.028	1.67	[1.10–2.54]	0.016
Children in families with 3 children (n_1_ = 4 823+7 378, n_2_ = 134+220)	1.58	[1.34–1.85]	<0.001	1.57	[1.33–1.86]	<0.001	1.80	[1.15–2.81]	0.010	2.24	[1.37–3.66]	0.001
Children in families with 4 children (n_1_ = 4 823+2 208, n_2_ = 134+81)	1.27	[1.00–1.60]	0.048	1.33	[1.04–1.70]	0.021	0.89	[0.51–1.55]	0.681	1.10	[0.61–1.99]	0.758

* Including first-born,

1 Adjusted for birth weight, birth year,

2 Adjusted for maternal age when giving birth, sex, maternal BMI (age 13 years),

3 Adjusted for father’s social class, n_1_ = number in the CSHRR, n_2_ = number in the DB.

**Table 3:** Odds ratios (OR) and 95% confidence intervals (CI) for odds of obesity in first-born children by family size.

	Copenhagen School Health Records Register (Obese: ≥95^th^ percentile)						Draft Board (Obese: BMI ≥31.00 kg/m^2^)					
	Crude			Adjusted^1,2^			Crude			Adjusted^1,3^		
First-born children* vs.	OR	95% CI	p-value	OR	95% CI	p-value	OR	95% CI	p-value	OR	95% CI	p-value
Any later born children (n_1_ = 12 035+12 469, n_2_ = 194+570)	0.73	[0.65–0.82]	0.006	0.89	[0.78–1.03]	0.121	0.73	[0.53–1.01]	0.057	0.81	[1.18–2.61]	0.209
Later born children in families with 2 children (n_1_ = 8 014+6128, n_2_ = 194+198)	0.67	[0.58–0.78]	<0.001	0.81	[0.68–0.96]	0.017	0.73	[0.48–1.11]	0.144	0.7	[1.10–2.54]	0.257
Later born children in families with 3 children (n_1_ = 3 044+4 334, n_2_ = 61+159)	0.85	[0.67–1.07]	0.169	1.05	[0.79–1.40]	0.724	0.84	[0.44–1.62]	0.606	0.85	[1.37–3.66]	0.658
Later born children in families with 4 children (n_1_ = 744+1 464, n_2_ = 9+72)	0.50	[0.32–0.79]	0.003	0.76	[0.44–1.33]	0.338	1.48	[0.37–5.96]	0.583	1.33	[0.61–1.99]	0.696

* Excluding only children.

1 Adjusted for birth weight, birth year,

2 Adjusted for maternal age when giving birth, sex, maternal BMI (age 13 years),

3 Adjusted for father’s social class. n_1_ = number in the CSHRR, n_2_ = number in the DB.

**Table 4:** Odds ratios (OR) and 95% confidence intervals (CI) for odds of obesity in last-born children by family size.

	Copenhagen School Health Records Register (Obese: ≥95^th^ percentile)						Draft Board (Obese: BMI ≥31.00 kg/m^2^)					
	Crude			Adjusted^1,2^			Crude			Adjusted^1,3^		
Last-born children* vs.	OR	95% CI	p-value	OR	95% CI	p-value	OR	95% CI	p-value	OR	95% CI	p-value
Any prior born children (n_1_ = 8 438+16 066, n_2_ = 361+403)	1.57	[1.40–1.77]	<0.001	1.33	[1.15–1.53]	0.001	1.36	[1.01–1.84]	0.043	1.32	[0.96–1.81]	0.087
Prior born children in families with 2 children (n_1_ = 6 128+8014, n_2_ = 198+194)	1.49	[1.29–1.72]	<0.001	1.24	[1.04–1.48]	0.016	1.37	[0.90–2.09]	0.144	1.29	[0.83–1.99]	0.257
Prior born children in families with 3 children (n_1_ = 1 842+5 536, n_2_ = 82+138)	1.63	[1.28–2.07]	<0.001	1.47	[1.07–2.00]	0.017	1.72	[0.96–3.09]	0.069	1.64	[0.86–3.13]	0.136
Prior born children in families with 4 children (n_1_ = 378+1 830, n_2_ = 36+45)	2.54	[1.71–3.78]	<0.001	1.93	[1.09–3.43]	0.025	1.02	[0.42–2.46]	0.960	1.07	[0.43–2.66]	0.885

* Excluding only children.

1 Adjusted for birth weight, birth year,

2 Adjusted for maternal age when giving birth, sex, maternal BMI (age 13 years),

3 Adjusted for father’s social class. n_1_ = number in the CSHRR, n_2_ = number in the DB.

All analyses were performed both as crude (unadjusted) analyses and adjusted with respect to the covariates as outlined and listed above. If not otherwise stated, all results presented below belong to the adjusted analysis. For the children, sex-stratified analyses were performed; the results were essentially the same, thus, only the combined, sex-adjusted analyses are presented. Furthermore, we found no interaction with either maternal age or maternal BMI, and they were thus included in the childhood analyses as described above. The birth spacing covariate was used only for supplementary analyses.

In supplemental analyses odds of obesity in second vs. third born children and in third vs. fourth born children were investigated. Since birth weight changes with higher birth order [20], birth weight is an intermediate variable on the causal pathway between birth order and obesity. We investigated interactions between the exposure variables and birth weight in three groups (<2500 g, 2500–4000 g, >4000 g). No significant interactions with birth weight were observed in any of the associations investigated. Yet, interaction analyses of obesity risk in only vs. any later born children tended to be significant in childhood and thus analyses stratified by birth weight in three groups were performed here as well as sensitivity analyses only including those with a birth weight between 2500–4000 g. We used similar stratification in the young men, but the size of this cohort with only 27 subjects having a birth weight below 2500 g made the stratification analyses impossible in this cohort. Linear trend by increasing family size were tested by inclusion of interaction terms to the models with either only, first- and last-born children as the dichotomous exposures multiplied with family size (1–4). Also, birth spacing between siblings was investigated as a possible explanatory variable in the children.

Since recommendations for measuring children’s BMI were changed in Copenhagen during the study period, a validation of the CSHRR was performed with comparison of BMI distributions between subjects who only had a BMI measurement at age 7 years available and those who had measurements available at both age 7 and 13 years, by ten years intervals of birth year.

In order to allow for intra-group correlation caused by having the same mother, we included a robust SE cluster function in all our regression analyses of the children. The estimated odds ratios (OR) are presented with 95% confidence intervals (CI). P-values of less than 0.05 were considered significant. All statistical analyses were performed using Stata version 9.2 and 11.0 (Stata Corporation, College Station, Texas; www.stata.com).

### Ethics Statement

The Danish Data Protection Agency has approved the use of the cohorts for this study (according to Danish law, ethical approval is not required for purely register-based studies).

## Results

The children was characterized by a median BMI at age 13 years of 25.3 kg/m^2^ in the obese group (BMI ≥95^th^ percentile) and of 18.0 kg/m^2^ in the remaining cohort (BMI <95^th^ percentile) (Table 1). The obese children more often had no siblings; they were on average born in later years and had higher birth weight; they were born to older mothers and by mothers with higher BMI at age 13 years. In addition, increased birth spacing between siblings was observed. The distributions of family size by birth cohort periods changed slightly with 16.4% and 18.3% being only children in 1952–1970 and 1971–1989, respectively. Also, the prevalence of families with four children was quite stable: 7.8% and 7.5%, respectively.

In the young men the median BMI at median age of 19 years was 32.7 kg/m^2^ in the case group versus 21.3 kg/m^2^ in the control group (Table 1). We observed no differences in birth weight or year of birth between the obese and the controls, respectively. The group of obese young men more often had no siblings, as also found in the children. The proportion of young men of unskilled/semiskilled fathers was higher in the case group compared with the control group. Associations between family size, birth order and obesity in the two study populations are found in Table 2, 3, 4, in which both the crude and adjusted measures are shown.

### Only Children

In childhood, being an only child significantly increased the odds of being obese (Figure 2) both when compared with any later born children OR 1.39 (95% CI: 1.19–1.61) and with children in a family with two, three or four children, respectively (Table 2, test for trend by increasing family size p = 0.005). Most of the associations became stronger after adjustments, and only children compared with children with two siblings (family size of three) showed the highest estimated risk of obesity with an adjusted OR of 1.57 (95% CI: 1.33–1.86).

Analyses stratified by birth weight showed that only children with a birth weight below 2500 g compared with any later born children (n = 1 132) had an OR of obesity of 2.41 (95% CI: 1.15–5.02). In the young men, very similar results were observed, with the highest odds ratio of obesity in men being only children compared with those having two siblings (Table 2). Men being only children compared with men being later born having any siblings had an adjusted OR of 1.64 (95% CI: 1.08–2.49) of obesity around age 19 years (Figure 2).

### First-born Children

In childhood, first-born children (excluding only children) were found to have a lower odds ratio of obesity than any later born children, but although the tendency remained, it did not stay significant after adjustments. No significant linear trend across increasing family size was observed; p = 0.26. Compared with the second, third or fourth born child in families of two, three or four children, the first-born child also generally showed a tendency towards lower odds of being obese. After adjustment, only the association observed in two child families remained significant (Table 3). In first-born children having a birth weight below 2500 g, the odds of obesity was not significantly higher compared with any later born with a birth weight below 2500 g (n = 1 536), OR = 1.38 (95% CI: 0.67–2.84), but this OR still showed some tendency of being increased as compared with the corresponding non-stratified statistic (presented in Table 3). No significant association between first-born status and odds of being obese was found in the young men (Table 3). Only children, compared to first-born children, had a significantly higher odds of obesity both in childhood as well as in the young adult men with adjusted ORs of 1.54 (95% CI: 1.31–1.80) and 1.97 (95% CI: 1.25–3.10), respectively (Figure 2).

### Last-born Children

Last-born children (excluding only children) were significantly more likely to be obese in childhood (Table 4). These associations were attenuated, but remained significant after adjustment with an OR of 1.93 (95% CI: 1.09–3.43) within families of four children. No significant linear trend across increasing family size was observed; p = 0.5. However, last-born children showed lower odds of obesity than only children: only children’s odds of being obese exceeded the odds in last-born children with OR = 1.25 (95% CI: 1.06–1.47) adjusted for relevant confounders. This was significant in childhood only (Figure 2), but the estimates were similar in the young men, OR = 1.43 (95% CI: 0.93–2.20). Analyses of birth spacing between siblings in childhood showed no overall differences in last-born children by combinations of family size and birth order. Yet, when comparing obese and non-obese last-born children by increasing family size we found significantly higher birth spacing in the obese last-born children than in the non-obese last-born children in two (1721 vs. 1412 days; p<0.001), three (1802 vs. 1531 days; p = 0.050) and four child families (1872 vs. 1303 days; p = 0.026).

No differences in odds of obesity in second vs. third born as well as third vs. fourth born children were found (not shown). Sensitivity analyses only including children with a birth weight between 2500–4000 g showed marginal changes in all OR risk estimates (not shown). Finally, the comparison of BMI distributions between subjects only with age 7 years BMI measurements and those with measurements both at age 7 and 13 years by ten years intervals of birth year revealed no differences.

## Discussion

The aims of this study were to explore if, and how, family size and birth order are associated with obesity. We found that only children had higher odds of obesity than children with siblings, and this was not explained by their status of being first-born. Furthermore, last-born children were also more often obese when compared with prior born children, and a comparison of only and lastborn children showed a significant difference in odds of obesity for the adjusted CSHRR analysis only.

The reason why obesity risk differs by birth order and family size is unknown, but differences in fetal nutrition and changes in this in successive pregnancies, reflected in e.g. birth weight (The Developmental Origins Hypothesis), have been put forward [8], [21]. If this was the explanation for the observed increased risk in only children, we would expect first-born children to have the same risk, unless mothers who only have one child are affected by biological conditions associated both with the number of children they subsequently get as well as their child’s obesity risk. Such a condition could be pre-pregnancy overweight. In relation to this, we observed similar BMIs at age 13 years in mothers of only children compared with mothers with two and three children in the CSHRR. The observation of the high OR of obesity, found when comparing only and later born children of low birth weight (below 2500 g), is interesting and needs further investigation. In addition, the analogous comparison was not significant for first-borns of low birth weight, but still showed a clearly indicative increase as compared with the non-stratified case.

The reason why last-born children are at increased risk of obesity may have developmental origin such as increasing maternal age and pre-pregnancy BMI for every successive pregnancy. We did take these factors partly into account by adjusting for maternal age as well as maternal BMI at age 13 years although an influence by increasing maternal weight for every successive pregnancy is possible. In the supplemental analyses, we found no significant differences in obesity odds when comparing second vs. third born children as well as in third vs. fourth born children, which we would have expected if increasing maternal weight with increasing birth order in general were the explanation for the observed higher odds in last-born children. Thus, other factors, possibly of psychosocial origin, may explain the associations. The finding of increased birth spacing between siblings, as observed in obese children in general as well as in obese last-born children is interesting. It is possible that maternal behavioral factors influence this, equally as in the relation to the observed higher risk in only children. However, further investigation is needed to clarify the origin of these findings. Bruch *et al.* described that obese children often were only or last-born children, and their mothers often displayed ambivalence in feelings towards them [2]. Only children were not found to be at increased risk in a study by Lissau *et al.*, who instead found that neglected children were at greatly increased risk of obesity [22]. In addition, insecure attachment style [23], maltreatment [24] and family stress [25] have been associated with childhood overweight, but whether these factors differ along with family size and birth order remain to be elucidated.

Previous studies have indicated that having a higher birth order increases the risk of obesity. In our cohorts, however, increasing odds of being obese was primarily attributed to last-born child status in a family, as described by Bruch [2]. It has previously been observed that last-born children have psychological characteristics like those of only children [26]. Whether this influences their obesity risk is not known.

We did not find any sex differences in the associations in children. In addition, since we only studied young adult men, it is unknown whether there are sex differences in the associations in adulthood. The consistency of associations in children as well as in young adult men is worth noticing, irrespective of the drivers behind these. Family sizes did change during our study period with, for instance, the prevalence of only children increasing from 16.4 per cent in 1952–70 to 18.3 per cent in 1971–89 in the CSHRR. Thus, changes in family size may have contributed to the development of the obesity epidemic [27]. Research aiming at identifying causes, whether psychological or biological, of the increased obesity risk in only and last-born children is warranted.

We carried out many analyses due to the combined exposure structure of family size and birth order. Yet, even with a Bonferroni multiple testing corrections for 100 analyses (approximately the total number of analyses performed here), the observed higher risk in e.g. only children compared with children with siblings would still reach significance (uncorrected p-value = 0.00002; Bonferroni corrected p-value = 0.00002*100 = 0.002) in the CSHRR, as would the observed higher risk in last-born children compared to prior born children (uncorrected p-value: p = 0.0001; Bonferroni corrected p-value = 0.0001*100 = 0.01).

This study included two prospective study populations without selection by social class. The size of the CSHRR is a major strength, and the fact that we could replicate our findings in the young men, which due to the nested design actually represents a very large population (approximately 58 000) [13], further adds to the trustworthiness of findings. Both cohorts had objective measures of height and weight, and problems with misreporting were therefore avoided [28]. In the young adult men the criterion used to identify obesity later turned out to correspond to a BMI ≥31.00. Due to the case-cohort sampling design, it would require a manual review of all the almost 400,000 old paper records to change the cut-off point to the nowadays commonly used threshold of BMI ≥30.00. However, this was not possible. Yet, we do not suspect that using a cut-point of BMI ≥31 instead of BMI ≥30 would have changed any of the associations or conclusions in this study. The information on exposures and outcome in the children was obtained from large registers [15]. Even though recommendations for measuring school children at school exit changed in year 1983, the comparison of the included and not included population revealed no differences. Therefore, we suspect no systematic bias in the BMIs of the included study population. The Fertility Database was established in year 1980 and included every child living with its mother at that time. Approximately 70% of children born between years 1952–60 have a connection to their mother, whereas almost every mother-child pair (96.7%) have been connected between birth years 1960–89 [29].

Analyses were adjusted for important confounders such as the mothers pre-pregnancy BMI in the analysis of the children and social class of the young men’s fathers, as well as other known obesity risk factors such as birth weight and year of birth [1]. Our study, however, does have some limitations. First, the individuals in our cohorts were born between 1930 and 1989; hence, family dynamics may have changed since this period and may therefore not be completely comparable to present-day societies [30]. Second, obesity in young adulthood was only investigated in male subjects. Third, numbers of siblings were noted at first school health examination at age six or seven years for the young men and may have changed afterwards. For the children, birth order and family size were obtained from national registers, and with individuals being at least 22 years old at present, it is unlikely that family size will increase further. In any case, this will not influence obesity status in childhood among the siblings born earlier. Family size was obtained through biologically related mothers, which means that paternal-origin, half siblings or social siblings may be present, if children from previous marriages were living with their fathers permanently, and he then found a new spouse (with or without a child). Yet, at the time it was not common for fathers to have full custody (which means that the child would only stay with him every second or third weekend) [31]. Therefore, this is not seen as a direct bias of the only child status in a family obtained through mothers. Maternal obesity was determined from a measurement at age 13 years, and later BMI changes, departing from the tracking, are possible [32]. On the other hand, using BMI at age 13 years makes it possible to avoid self-reported weight and height, which is known to be uncertain [28]. Finally, residual confounding such as the influence from maternal preeclampsia or smoking status during pregnancy may still be present. Future cohort studies with this information available should look into the possible association with especially the obesity observed in only children.

In conclusion, being an only or last-born child in a family is associated with higher odds of obesity in both childhood and young adulthood. Being a first-born child in general is, however, not associated with higher odds of obesity. Identification of the mechanism behind the increased obesity risk in only and last-born children may provide leads to targeted prevention of obesity in these specific groups at early ages.
